# Fragments of Bacterial Endoglycosidase S and Immunoglobulin G Reveal Subdomains of Each That Contribute to Deglycosylation*

**DOI:** 10.1074/jbc.M113.532812

**Published:** 2014-03-25

**Authors:** Emma V. Dixon, Jolyon K. Claridge, David J. Harvey, Kavitha Baruah, Xiaojie Yu, Snezana Vesiljevic, Susan Mattick, Laura K. Pritchard, Benjamin Krishna, Christopher N. Scanlan, Jason R. Schnell, Matthew K. Higgins, Nicole Zitzmann, Max Crispin

**Affiliations:** From the ‡ Oxford Glycobiology Institute, Department of Biochemistry and; § Department of Biochemistry, University of Oxford, South Parks Rd., Oxford OX1 3QU, United Kingdom, and; ¶ School of Life Sciences, University of Warwick, Gibbet Hill Campus, Coventry, CV4 7AL, United Kingdom

**Keywords:** Antibody, Antibody engineering, Enzyme Mechanism, Glycoprotein, Glycosylation

## Abstract

Endoglycosidase S (EndoS) is a glycoside-hydrolase secreted by the bacterium *Streptococcus pyogenes*. EndoS preferentially hydrolyzes the *N*-linked glycans from the Fc region of IgG during infection. This hydrolysis impedes Fc functionality and contributes to the immune evasion strategy of *S. pyogenes*. Here, we investigate the mechanism of human serum IgG deactivation by EndoS. We expressed fragments of IgG1 and demonstrated that EndoS was catalytically active against all of them including the isolated CH2 domain of the Fc domain. Similarly, we sought to investigate which domains within EndoS could contribute to activity. Bioinformatics analysis of the domain organization of EndoS confirmed the previous predictions of a chitinase domain and leucine-rich repeat but also revealed a putative carbohydrate binding module (CBM) followed by a C-terminal region. Using expressed fragments of EndoS, circular dichroism of the isolated CBM, and a CBM-C-terminal region fusion revealed folded domains dominated by β sheet and α helical structure, respectively. Nuclear magnetic resonance analysis of the CBM with monosaccharides was suggestive of carbohydrate binding functionality. Functional analysis of truncations of EndoS revealed that, whereas the C-terminal of EndoS is dispensable for activity, its deletion impedes the hydrolysis of IgG glycans.

## Teaser

**Background:** Endoglycosidase S (EndoS) is an immune evasion factor from *Streptococcus pyogenes* that impedes IgG effector functions by deglycosylation.

**Results:** Analysis of fragments of enzyme and substrate identify components of each that contribute to catalysis.

**Conclusion:** Regions outside of the catalytic domain of EndoS contribute to IgG deglycosylation, and catalysis does not require all antibody subunits.

**Significance:** Engineering of EndoS specificity requires consideration of both catalytic and non-catalytic regions.

## Introduction

EndoS^4^ is a secreted glycoside hydrolase produced by the group A *Streptococcus pyogenes*, which shows specific endoglycosidase activity against human immunoglobulin G (IgG) (1). Deglycosylation impairs the effector functions of IgG, contributing to the immune evasion strategy of *S. pyogenes*.

IgG is a heterodimeric protein comprising two covalently linked subunits, each containing a heavy chain and a light chain. This multidomain structure contributes to the two key functions of IgG. First, IgG has antigen binding capability manifested in the variable Fab domains of the protein. Second, IgG has the capacity to elicit immunological responses through the conserved Fc domain. These Fc-mediated responses include binding to various Fc γ receptors as well as activation of the complement pathway (for review, see Ref. 2). Within the Fc domain, there is an *N*-linked glycosylation site on the IgG heavy chain. This conserved glycan contributes heavily to the structural integrity of the IgG Fc region where deglycosylation disrupts the effector functions of IgG (3–5). EndoS-mediated deglycosylation of the IgG heavy chain causes a disruption of Fc receptor binding. In the context of infection, this results in impaired killing of *S. pyogenes* through opsonophagocytosis. This supports the notion of EndoS involvement in *S. pyogenes* immune evasion (6).

Specifically, EndoS cleaves the β1→4 linkage within the di-*N*-acetylchitobiose core of the *N*-linked glycan present at Asn-297 of the IgG CH2 domain (1). EndoS also displays activity against glycans free in solution. In this context EndoS shows specificity for complex biantennary-type glycans and as such has gained interest for use as a specific transglycosidase for the chemoenzymatic synthesis of antibody glycoforms (7, 49).

EndoS is one of multiple IgG-targeting immune evasion factors secreted by *S. pyogenes* during infection. *S. pyogenes* also secretes the cysteine proteases SpeB and IdeS that cleave at the hinge region of IgG (for review, see Ref. 8). In addition to these proteases, a secondary endoglycosidase EndoS2 is present in virulent M49 strains (9). In addition to the activity of SpeB against IgG, this enzyme displays proteolytic activity against EndoS at position 446, rendering it catalytically inactive (10). This SpeB-mediated EndoS inactivation is perhaps a self-regulatory mechanism on the part of *S. pyogenes*. Alternatively, it may be a result of the relatively low specificity of SpeB. IdeS is a more specific protease, which as of yet does not show proteolytic activity against any tested substrate other than IgG. For this reason IdeS is actively being explored as a tool for treatment of IgG-mediated immune diseases (11, 12).

In parallel and in combination to IdeS, the ability of EndoS to specifically deglycosylate IgG has led to exploration of its activity in the treatment of immunological disorders (13, 14). EndoS has been successful in *in vivo* studies for the treatment of model systems of rheumatoid arthritis, experimental glomerulonephritis, and systemic lupus erythematosus (11, 15–17).

EndoS is also of interest for use in enhancing monoclonal antibody receptor interactions (5). In this application IgG glycoforms less susceptible to EndoS could maintain Fc γ receptor binding activity in the presence of competing serum IgG when treated with EndoS. Despite the success of engineering more resistant IgG glycoforms for this application, engineering protein mutations, which confer EndoS resistance to IgG, would also assist in the development of monoclonal antibodies suitable for this application. In addition, monoclonal antibodies resistant to *S. pyogenes* immune evasion factors, principally EndoS and the IdeS protease, might offer a further route to the treatment of *S. pyogenes* infections. Understanding and characterizing the interaction between EndoS and IgG is an important step in the development of these synthetic and therapeutic applications.

Homology modeling has given insight into the overall topology of EndoS (1, 10). A chitinase domain dominates the N-terminal region of EndoS and displays homology to family 18 glycoside hydrolases. Mutagenesis of the proposed catalytic residue from this domain resulted in an apparent loss of activity, supporting the predicted assignment of this region as a chitinase domain (2, 10). Downstream of the chitinase domain, EndoS contains a leucine-rich repeat (LRR). LRRs are structurally well characterized and are commonly involved in protein-protein interactions (for review, see Refs. 3, 4, and 18). Considering that EndoS is inactive against denatured IgG, protein-protein as well as protein-glycan interactions are likely to play a role in activity (5, 19). The LRR may be involved in these protein-specific IgG-EndoS interactions and contribute to activity in this way.

In an effort to characterize the IgG-EndoS interaction, we have analyzed truncated domains of IgG and subsequently the ability of EndoS to deglycosylate these domains. Furthermore, we have probed the amino acid sequence of EndoS to better characterize the C-terminal region of the protein, and we report the presence of a carbohydrate binding module (CBM).

## EXPERIMENTAL PROCEDURES

### 

#### 

##### Cloning and Expression

The constructs for IgG1 Fc, CH2-H, and CH2 were cloned for recombinant expression in mammalian cells. The gene for human IgG1 Fc encoding residues 224–446 (SWISS-PROT accession number P01857.1) was cloned into the mammalian expression vector, pHLSec, as described previously (6, 20). Using the same IgG1 Fc sequence as a template, a CH2-H construct was designed to contain the hinge region and CH2 domain of IgG1 Fc (residues 224–338), and a CH2 construct was made to solely encompass the CH2 domain of IgG1 Fc (residues 231–338). Both the CH2-H and CH2 genes were synthesized by GeneArt (Invitrogen) to contain additional 5′ and 3′ sequences to allow compatibility with the In-Fusion cloning system (Clontech) and were cloned as such into the vector pHLSec.

The Fc, CH2-H, and CH2 glycoforms were transiently expressed in HEK 293T cells (ATCC number CRL-1573) as described previously (1, 21). Briefly, cells were grown in standard T225 flasks (Corning) at 37 °C in a humidified incubator containing 5% CO_2_. Cells were cultured in Dulbecco's modified Eagle's medium (DMEM; Invitrogen) supplemented with 10% fetal bovine serum and 1% penicillin/streptomycin. For transient expression, endotoxin-free plasmid DNA containing the relevant construct was mixed with polyethyleneimine at a mass ratio of 1:1.5 in DMEM containing 1% penicillin/streptomycin. Cells were cultured to 90% confluence before being transfected with the DNA:polyethyleneimine mixture. The cells were grown for a further 4 days in DMEM, 1% fetal bovine serum, and 1% penicillin/streptomycin at 37 °C, 5% CO_2_. Full-length IgG from human serum was purchased from Sigma.

A plasmid containing an N-terminally glutathione *S*-transferase (GST)-tagged construct of the full-length EndoS protein was kindly provided by Prof. Ben Davis (University of Oxford) (7). Briefly, *S. pyogenes* serotype M1 *ndoS* nucleotide sequence (GenBank^TM^ accession number AF296340) was codon-optimized for *Escherichia coli* expression. The optimized gene was then synthesized by GenScript to contain both 3′ BamHI and 5′ NotI restriction endonuclease sites. Using these sites, the resultant gene was cloned into the expression vector pGEX-4T-1 (GE Healthcare).

The pGEX-4T-1-*ndoS* vector was used as a template for generating the various EndoS domain constructs. The CBM-KO construct was generated via overlap PCR to remove residues 761–924. The remaining constructs, ChitLRR (residues 1–760), CBM (residues 761–924), and CBM-CT (residues 761–995), were amplified by PCR to be cloned into bacterial expression vectors. ChitLRR was cloned into pGEX-4T-1 (GE Healthcare), whereas the CBM-KO, CBM, and CBM-CT constructs were cloned into Champion^TM^ pET303 (Invitrogen).

All EndoS constructs were transformed into *E. coli* BL21 (DE3) SOLO^TM^ cells (Lucigen) following the manufacturer's instructions. EndoS and ChitLRR were expressed as N-terminal GST fusions. Using baffled 2-liter conical flasks, a 500-ml culture of cells was grown in Miller lysogeny broth containing carbenicillin (100 μg/ml) at 37 °C with shaking (250 rpm). Upon reaching an optical density at 600 nm (*A*_600_) of 0.6–0.7, the cells were induced with 1 mm isopropyl β-d-1-thiogalactopyranoside. The cells were then incubated for a further 4 h at 37 °C at 250 rpm. CBM-KO, CBM, and CBM-CT were all expressed with a C-terminal hexahistidine tag. For these constructs, a 500-ml culture of cells was grown in 2-liter baffled conical flasks using Magic Media^TM^ (Invitrogen) containing carbenicillin (100 μg/ml). Cells were grown at 37 °C with shaking (250 rpm) to an *A*_600_ of 0.6–0.7 at which point the temperature was reduced to 25 °C for a further 24 h of growth.

Expression of isotopically labeled ^15^N-CBM was achieved using a modified recipe for C-75052 media (8, 22). Here, the recipe was amended to contain no α-lactose or glycerol and was supplemented with 0.5% glucose. A 500-ml culture was grown in modified C-75052 containing carbenicillin (100 μg/ml) at 37 °C with shaking (250 rpm). The cells were cultured to an *A*_600_ 0.7–0.8 before being induced with 1 mm β-d-1-thiogalactopyranoside. The cells were then incubated at 25 °C with shaking (250 rpm) for a further 20 h post induction. For all constructs, cells were harvested by centrifugation (6000 × *g* for 15 min) and frozen at −80 °C before lysis and purification.

##### Purification of IgG Domains

For Fc, CH2-H, and CH2, cell culture supernatant was decanted from culture flasks 4 days post-transfection and centrifuged at 2000 × *g* for 5 min to pellet any residual cells. The supernatant was then passed through a 0.22-μm filter and diluted 1:1 with binding buffer (PBS, 10 mm HEPES, pH 8.0).

The protein of interest was then purified by immobilized metal affinity chromatography using HisPur^TM^ Ni-NTA resin (ThermoScientific). Eluted fractions containing the target protein were concentrated using Vivaspin20 concentrators (10-kDa molecular mass cutoff for Fc, 3-kDa molecular mass cutoff for CH2-H and CH2) for further purification by size exclusion chromatography. Samples were applied to a pre-equilibrated Superdex200 HiPrep 16/60 (Fc) or Superdex75 HiPrep 16/60 (CH2-H, CH2) column. Protein was eluted with 10 mm HEPES, pH 8.0, 150 mm NaCl. The absorbance at 280 nm (*A*_280_) of the eluate was monitored to determine the presence of protein. Protein purity was assessed by SDS-PAGE.

##### Purification of EndoS Domains

Pelleted cells were resuspended in PBS containing lysozyme (Sigma), DNase I (Roche Applied Science), and protease inhibitors (Complete EDTA-free; Roche Applied Science). Cells were then mechanically lysed using a single pass through a cell disruptor at 30 kp.s.i. (Constant Systems). Cell lysate was clarified by centrifugation at 30,000 × *g* for 30 min. The supernatant was passed through a 0.22-μm filter before further purification.

##### Purification of EndoS and ChitLRR

GST fusion was purified from clarified supernatant using a GSTPrep^TM^ FF 16/10 column (GE Healthcare) as previously described (7, 9). Fractions containing putative GST fusion were pooled, and total protein content was measured by bicinchoninic acid assay (10, 23) (Pierce). Thrombin (GE Healthcare) was then added at 1 units/0.1 mg of protein and incubated at room temperature for 24 h to remove the GST tag.

The cleaved sample was then dialyzed into 20 mm Tris, pH 8.0, 10 mm NaCl in preparation for anion exchange chromatography using a HiPrep Q HP column (GE Healthcare). The sample was purified from contaminants using buffer A (20 mm Tris, pH 8.0) and an increasing gradient of buffer B (20 mm Tris pH 8.0, 1 m NaCl). Fractions containing EndoS or ChitLRR were pooled and concentrated using a Vivaspin20 concentrator (10-kDa molecular mass cutoff). The sample was then purified by size exclusion chromatography using a HiPrep SephacrylS200 16/60 column (GE Healthcare) pre-equilibrated with 10 mm HEPES, pH 8.0, 150 mm NaCl.

##### Purification of CBM-KO, CBM, and CBM-CT

His-tagged constructs were purified from clarified supernatant using HisPur^TM^ Ni-NTA resin (ThermoScientific). Eluted fractions containing the target protein were concentrated using Vivaspin20 concentrators (10-kDa molecular mass cutoff) for further purification. At this stage CBM-KO was subjected to an additional anion-exchange step using a HiPrep Q HP column (GE Healthcare). CBM-KO was dialyzed into 20 mm Tris, pH 8.0, 20 mm NaCl and then purified using a gradient of buffer A and B as described above. CBM-KO, CBM, and CBM-CT were then purified by size exclusion chromatography. Samples were applied to a pre-equilibrated Superdex200 HiPrep 16/60 (CBM-KO) or Superdex75 HiPrep 16/60 (CBM, CBM-CT) column. Protein was eluted with 10 mm HEPES, pH 8.0, 150 mm NaCl (CBM-KO), or 20 mm Tris, pH 7.5, 150 mm NaCl (CBM, CBM-CT).

##### Enzymatic Release of N-Linked Glycans

For release of *N*-linked glycans by EndoS, reactions were performed in PBS at a molar ratio of 1:10 EndoS to substrate. The reaction was allowed to proceed for 24 h. Release of the total or remaining pool of *N*-linked glycans was carried out in solution using protein *N*-glycosidase F (PNGase F; New England Biolabs) following the manufacturer's instructions. Enzymatically released glycans were purified from enzyme reactions using a Ludger CleanTM Post-Exoglycosidase Clean-up Plate (Ludger). PNGase F release of *N*-linked glycans from SDS-PAGE gel bands was performed as described previously (20, 24).

##### Analysis of Released N-Linked Glycans

For HPLC analysis, glycans were fluorescently labeled using LudgerTag^TM^ 2AA (2-aminobenzoic acid) glycan labeling kit (Ludger). To remove excess dye, labeled glycans were purified with LudgerClean^TM^ T1 Glycan Cleanup Cartridges using a vacuum manifold (Ludger). Eluted glycans were then analyzed by HPLC using a LudgerSep^TM^ N2 High Resolution Amide column employing a gradient of solvent A (1.25 mm ammonium formate, pH 4.4) and solvent B (acetonitrile). The sample was solvated in 35% MilliQ water, 65% B and loaded onto the column pre-equilibrated with 35% A. The gradient was increased from 35 to 46% A over 22 min at 0.7 ml/min and was further increased from 46 to 50% B over 2 min at 0.7 ml/min. The gradient was then set at 100% A for 2 min at 0.7 ml/min. Fluorescence was detected using an excitation wavelength of 360 nm and a detection wavelength of 425 nm.

The identity of the glycans was confirmed by MALDI-TOF MS and negative ion electrospray ionization-S/MS (11, 12, 25) using unlabeled glycans cleaned with a Nafion 117® membrane (13, 14, 26). MALDI-TOF spectra were recorded with a Shimadzu-Kratos TOF^2^ mass spectrometer (Shimadzu Biotech, Manchester, UK) with 2,5-dihydroxybenzoic acid as the matrix.

Nanoelectrospray mass spectrometry was performed with a Waters Synapt G2 ion mobility quadrupole-time-of-flight instrument. Samples in 1:1 (v/v) methanol:water containing 0.5 mm ammonium phosphate (to ensure maximum formation of phosphate adducts) were infused through Waters thin-wall nanospray capillaries. The ion source conditions were: temperature, 80 °C; infusion needle potential, 1.2 kV; cone voltage 100 V. The traveling-wave ion-mobility cell (nitrogen) was operated with a wave velocity of 450 m s^−1^ and a wave height of 40 V. For MS/MS data acquisition, the parent ion was selected at low resolution (about 4 *m*/*z* mass window) and fragmented with argon. The voltage on the collision cell was adjusted with mass to give an even distribution of fragment ions across the mass scale. Typical values were 80–120 V. Spectra (2 s scans) were acquired with a digitization rate of 4 GHz and accumulated until a satisfactory signal:noise ratio had been obtained. Other operating voltages were as recommended by the manufacturer. Instrument control, data acquisition, and processing were performed with MassLynx software Version 4.1, and Waters DriftScope software was used to extract singly charged glycan ions from the total profile and to reject MS/MS fragment ions that were not associated with the target glycan (11, 15–17, 27). Glycan fragments were labeled according to the scheme proposed by Domon and Costello (28).

##### SDS-PAGE Analysis

SDS-PAGE was carried out using Novex NuPAGE Bis-Tris 4–12% pre-cast gels. Electrophoresis was performed in NuPAGE MES SDS running buffer (Invitrogen) using an XCell SureLock^TM^ Electrophoresis Cell (Invitrogen). All samples were mixed with NuPAGE LDS Sample Buffer (Invitrogen) and reduced using dithiothreitol then boiled at 95 °C for 2 min before electrophoresis. In the case of the IgG fragments, samples were not reduced or boiled but were mixed with sample buffer before electrophoresis.

##### Bioinformatic Analysis of EndoS

Using the online server for the program FUGUE (29), the protein sequence for EndoS was analyzed for regions of homologous protein structure. The three-dimensional reconstructions of FUGUE-generated models were visualized using VMD (30).

##### Circular Dichroism (CD)

CD spectra were collected using a Jasco J-815 spectrometer (Jasco). Protein solutions were buffered with 10 mm sodium phosphate, pH 7.2. Protein concentrations were calculated by measuring the absorbance at 280 nm in conjunction with the predicted extinction coefficient of 30,940 m^−1^cm^−1^ for both CBM and CBM-CT. The extinction coefficient was calculated using Protparam from the ExPASY server (31), which employs an Edelhoch based method (32–34). Spectra were acquired using a quartz cuvette with a 0.1-cm path length. The spectra were collected between 260–180 nm at 50 nm min^−1^ at 0.5-nm increments with a response time of 0.5 s and a data pitch of 0.5 nm. Base lines were collected in the same manner using 10 mm sodium phosphate buffer, pH 7.2. Spectra were averaged over 4 scans then corrected for solvent background and smoothed using a Savitzky-Golay method (35). To determine secondary structure components, various deconvolution methods were applied to the data, which were accessed using the DICHROWEB server (36).

##### NMR Titrations

Purified ^15^N-CBM (0.15 mm) was titrated with increasing concentrations of d-galactose or l-fructose (62.5 mm to 2 m). All experiments were performed in 10 mm BisTris, pH 6.5, 15 mm NaCl at 37 °C. NMR spectra were collected on a Bruker Avance III 600 MHz spectrometer fitted with a TCI cryoprobe. ^1^H,^15^N heteronuclear single-quantum coherence (HSQC) spectra were acquired with 16 scans. Acquisition time and spectral width were 107 ms and 9579 Hz in the direct dimension (^1^H) and 66 ms and 1929 Hz in the indirect dimension (^15^N), respectively. Spectra were processed with NMRpipe (37) and visualized using CcpNmr analysis (38).

##### Isothermal Calorimetry

Isothermal calorimetry titrations were carried out using a MicroCal iTC200 (GE Healthcare). All measurements were carried out at 20 °C. Purified CBM was dialyzed extensively into PBS, and a stock 1 m d-galactose solution was made by dissolving the appropriate mass of d-galactose in the dialysate produced during the dialysis of the CBM. The CBM (1.4 mm) was titrated with repeated 2-μl injections of 42 mm d-galactose. For the heat of dilution controls CBM was titrated with PBS and PBS was titrated with d-galactose. Control experiments utilized identical concentrations to those used in the binding experiment.

## RESULTS

Considering the potential scope for EndoS use in both therapeutic and transglycosidase applications, we sought to understand how its apparent specificity is conferred and what factors might be important for EndoS deglycosidase activity toward IgG.

### 

#### 

##### Glycan Analysis of IgG Fragments

**FIGURE 1.**
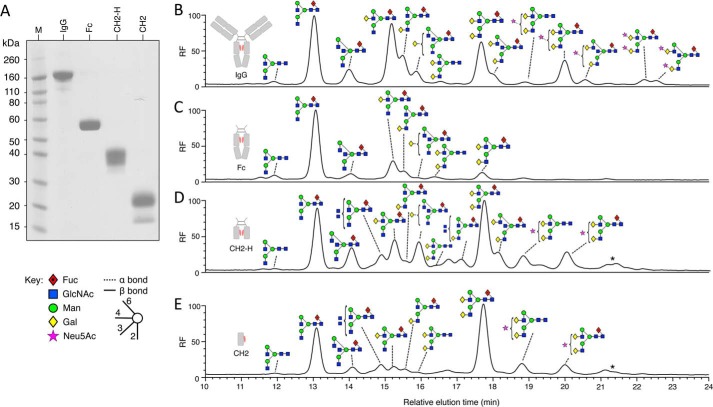
**Glycosylation profiling of serum IgG and recombinantly expressed IgG fragments.** Shown is SDS-PAGE analysis of serum IgG and purified IgG fragments (*A*). Shown is HPLC analysis of fluorescently labeled glycans released from serum IgG (*B*) and recombinantly expressed Fc (*C*), CH2-H (*D*), and CH2 (*E*) using PNGase F. Peaks were assigned using negative ion electrospray ionization-MS/MS and are labeled using Oxford glycan nomenclature (46) with the color scheme of the Consortium for Functional Glycomics as previously implemented (20, 47). The *star* denotes glycan with the composition Neu5Ac_2_Hex_5_GlcNAc_4_ (probably Neu5Ac_2_Gal_2_GlcNAc_2_Man_3_GlcNAc_2_), which was present in too low a quantity for fragmentation analysis. Fluorescence is reported as relative fluorescence units (*RF*).

As an initial step toward understanding the specificity of EndoS for IgG, we considered the minimal protein unit required for deglycosylation of IgG for EndoS. A number of constructs of the IgG Fc domain were designed to elucidate protein-protein interactions that might be important for EndoS activity against IgG. In addition to an already described Fc domain construct (20), further constructs were made to encompass the CH2 domain inclusive of the hinge region, capable of disulfide bonding (CH2-H), and the CH2 domain excluding the hinge region, predicted to exist as an isolated monomeric domain (CH2). These components could be transiently expressed in HEK 293T cells. All constructs were soluble and could be purified to >95% purity as judged by SDS-PAGE (Fig. 1*A*).

To assess the relevance of these constructs as targets for EndoS deglycosylation, we determined their overall pattern of glycosylation (Fig. 1, *B–E*). Glycan profiling of the total glycan pool from each of the fragments was carried out by PNGase F digestion of the purified fragments and comparing them to the glycosylation profile of human serum IgG. The glycan repertoire of the IgG fragments included the majority of the complex biantennary glycans routinely identified on human serum IgG. Compared with serum IgG, the IgG fragments had varying degrees of both galactosylated and bisected glycans. In addition, CH2-H and CH2 contained hybrid-type glycans as well as bisected triantennary or tetraantennary species. The expressed Fc fragment shows reduced glycan processing when compared with human serum IgG.

##### Identification of Glycans by Mass Spectrometry

**FIGURE 2.**
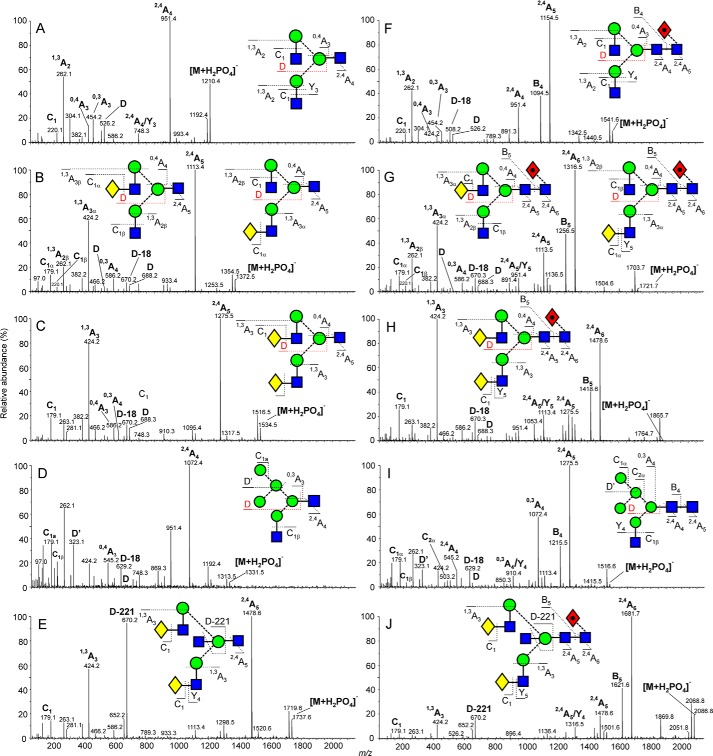
**Fragmentation analysis of released glycans from IgG.** Example fragmentation spectra are shown for EndoS released glycans (*A*, *B*, *C*, *D*, and *E*) and PNGase F released glycans (*F*, *G*, *H*, *I*, and *J*) from serum IgG. Fragmented glycans are represented using Oxford glycan nomenclature (46) combined with the color scheme of the Consortium for Functional Glycomics as previously implemented (20, 47). Fragments are labeled following the Domon and Costello nomenclature (28). The spectrum shown in *D* contains a contribution from ions (particularly the ions at *m*/*z* 262, 424, and 951) derived from another adduct of the glycan of composition Man_3_GlcNAc_3_ appearing at the same mass. Only partial separation of the ions from these compounds was achieved by ion mobility.

Compositions of the glycans in terms of isobaric residues such as hexose and *N*-acetylhexose (*N*-acetylhexosamine) were deduced from the masses measured by MALDI-TOF ([M+Na]^+^ ions) and negative ion nanoelectrospray ionization mass spectrometry ([M+H_2_PO_4_]^−^ and [M-H]^−^ ions). Detailed structures were determined by negative ion collision-induced decomposition in the transfer cell of the Synapt mass spectrometer (after mobility separation) (25) and were confirmed where possible by comparison with *N*-glycans released from well characterized glycoproteins such as ovalbumin and porcine thyroglobulin. Representative spectra are shown in Fig. 2 with glycans containing one core GlcNAc residue (released with EndoS) in the left-hand column and those with two GlcNAc residues (released with PNGase F) in the right-hand column. Diagnostic fragment ions discussed in earlier publications on PNGase F-released glycans were also prominent in the EndoS-released products.

The di-*N*-acetylchitobiose core region of the PNGase F-released glycans was characterized by the production of abundant ^2,4^A_R_, B_R_ and ^2,4^A_R-1_ ions (the subscript R in this discussion refers to the reducing terminal residue and R-1 to the penultimate residue; in Fig. 2, R and R-1 correspond to 4 and 5 or to 5 and 6 depending on the length of the antennae). Where a core fucose residue was present at the 6-position (Fig. 2, *spectra F*, *G*, *H*, and *J*), the ^2,4^A_R_ ion appeared at 405 rather than 259 mass units below that of the molecular ion. For the EndoS-released glycans, only a ^2,4^A_R_ ion was present.

The composition of the 6-antenna and, by difference, the 3-antenna, was defined by the mass of the D and D-18 ions that are formed by formal loss of the di-*N*-acetylchitobiose core and the 3-antenna, together in most cases with the ^0,3^A_R-2_ and ^0,4^A_R-2_ ions from the core branching mannose residue. The D and D-18 ions appear at *m*/*z* 526 and 508, respectively in the spectra of the biantennary glycans without galactose residues (*spectra A* and *F*) and at *m*/*z* 688 and 670 in those with galactose on the 6-antenna (*spectra C* and *H*). Where one galactose residue was present (*spectra B* and *G*) the presence of both sets of ions indicated the existence of isomers with galactose on either antenna. The ^0,3^A cross-ring cleavage ion defined the compositions of the antennae with the C_1_ ions defining the terminal residues. The hybrid glycans (*spectra D* and *I*) gave D and D-18 ions at *m*/*z* 647 and 629, respectively, with additional ^0,3^A_3_ and ^0,4^A_3_ ions characterizing the presence of the three mannose residues in the 6-antenna. The bisected glycans (*spectra E* and *J*) displayed very prominent ions at *m*/*z* 670 corresponding to D-221 (loss of the bisecting GlcNAc residue from the D ion). For more information on the interpretation of these negative ion collision-induced decomposition spectra see Harvey *et al.* (25).

##### Susceptibility of IgG Fragments to EndoS

**FIGURE 3.**
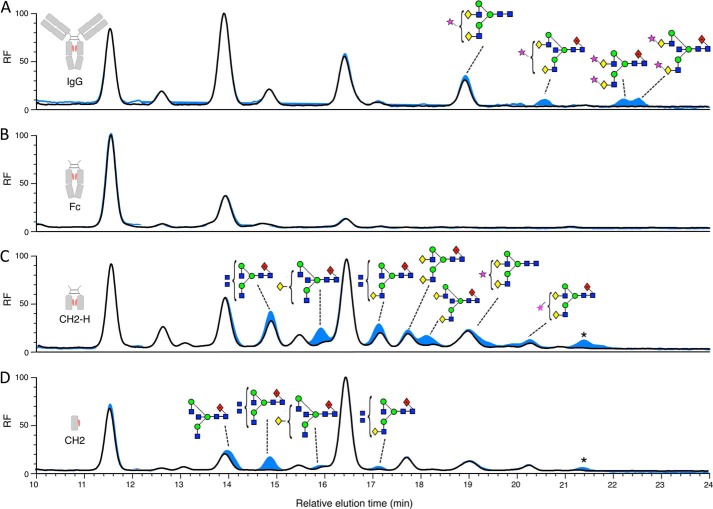
**Analysis of EndoS-released glycans from serum IgG and IgG fragments.** HPLC analysis of fluorescently labeled glycans released from serum IgG (*A*) and recombinantly expressed Fc (*B*), CH2-H (*C*), and CH2 (*D*) using EndoS (*black*) and sequential digest with EndoS then PNGase F (*blue*). Peaks were assigned using negative ion electrospray ionization-MS/MS. Glycans that remained after digest with EndoS, which were then cleaved as a result of sequential digest with PNGase F, are labeled using Oxford glycan nomenclature (46). The star denotes glycan with the composition Neu5Ac_2_Hex_5_GlcNAc_4_ (probably Neu5Ac_2_Gal_2_GlcNAc_2_Man_3_GlcNAc_2_) that was present in too low a quantity for fragmentation analysis. Fluorescence is reported as relative fluorescence units (*RF*).

Having shown that isolated domains of IgG1 could be expressed, we were interested in testing their susceptibility to EndoS deglycosylation. These experiments were conducted with an aim of determining if any protein-protein interactions involving missing domains were crucial to EndoS glycoside hydrolase activity against IgG1. In all cases, glycans could be hydrolyzed from the expressed IgG fragments (Fig. 3). In terms of human serum IgG, higher molecular weight sialylated IgG glycans appear less susceptible than the more abundant neutral glycans. The glycans of the Fc domain could be cleaved; however, small populations of uncleaved bisected biantennary glycans could be seen by MALDI-TOF MS analysis. The same observation was made for the CH2-H and CH2 glycoproteins where there was a population of uncleaved bisected biantennary glycans. In addition, the peaks representing either tetraantennary or bisected-triantennary glycans (in both galactosylated and agalactosylated forms) from CH2-H and CH2 appear more resistant to EndoS cleavage than other glycan species present on these isolated domains (Fig. 3, *C* and *D*).

##### EndoS Domain Architecture

**FIGURE 4.**
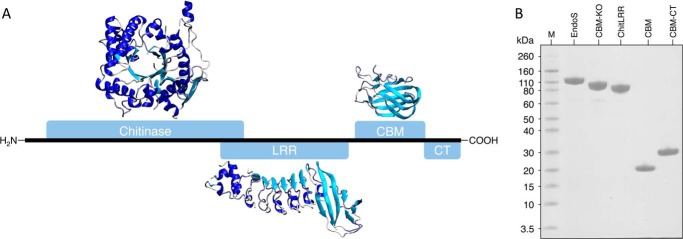
**EndoS domain architecture and purification of EndoS fragments.** The 995-amino acid sequence of EndoS is depicted as a *black line* with the domains annotated as *blue rectangles*, as identified by FUGUE analysis of the EndoS amino acid sequence. The model of the chitinase domain spans residues 48–500 and is based on an alignment of PDB accession numbers 1D2K, 1EDQ, 1E15, and 1E91 with a Z-score of 17.70. The LRR model includes residues 446–745 and is based on an alignment against PDB accession number 1M9S with a Z-score of 14.40. Finally, the CBM spans residues 771–924 where the model is based on an alignment to PDB accession numbers 1EUU and 1GOF and has a Z-score of 10.24. FUGUE was unable to detect any homologous structure to the CT region of EndoS from residues 925–995 (*A*). SDS-PAGE analysis of EndoS fragments after purification is shown (*B*).

Having ascertained that EndoS could hydrolyze the glycans from an isolated CH2 domain of human IgG1, we assessed the domain organization of EndoS in order to identify domains, which might influence enzymatic activity. Bioinformatic analysis of the EndoS amino acid sequence was carried out to search for structurally homologous domains and to help determine domain boundaries for construct design. Using the full-length amino acid sequence of EndoS as well as smaller sections of the sequence, the program FUGUE revealed further insight into the potential domain architecture of EndoS over and above a previously described homology model (10) (Fig. 4*A*). FUGUE was able to identify homologous structures for the majority of the amino acid sequence where the top scoring solutions (judged by highest Z-score) were all investigated. The top scoring solutions for the N-terminal chitinase domain and the LRR were generated from the same PDB entries that were recognized and used for the previously constructed homology model of EndoS (10). In addition, FUGUE identified a region in the C terminus of EndoS that has homology to a family of proteins called carbohydrate binding modules (CBMs) (for review, see Ref. 39). The final 100 residues of EndoS were unable to be assigned to any homologous structures using FUGUE.

To further investigate the putative CBM from EndoS, the results of the FUGUE analysis were used to guide the position of domain boundaries for the design of an isolated CBM construct. Further constructs were made to contain both the predicted CBM and the C-terminal region of EndoS (CBM-CT) and just the N-terminal chitinase and LRR portions of EndoS (ChitLRR). Finally, a CBM deletion mutant was cloned to ascertain the effect of removing this apparent domain from EndoS (CBM-KO).

The constructs described were cloned into bacterial expression vectors and could all be expressed in the soluble fraction using an *E. coli* expression system. Purification strategies for each of the EndoS constructs were developed so that each of the isolated domains could be purified to >95% purity as judged by SDS-PAGE (Fig. 4*B*). Notably, expression of the CBM and CBM-CT constructs gave pure protein yields of between 0.5 and 0.75 g per liter of culture.

**FIGURE 5.**
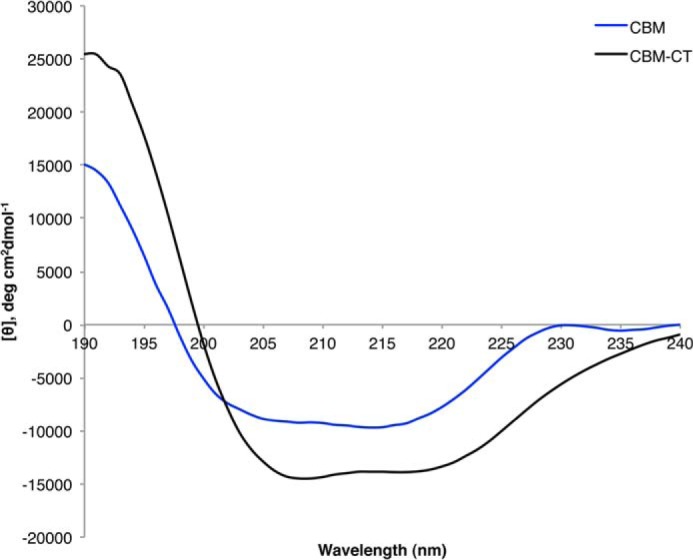
**Circular dichroism of the EndoS CBM and CBM-CT fragments.** Data are shown for circular dichroism measurements taken for CBM (*blue*) and CBM-CT (*black*) between 190–240 nm.

Investigation of the secondary structure of the CBM and CBM-CT fusion using CD supported the bioinformatics prediction of a primarily β-sheet CBM in the C terminus of EndoS (Fig. 5 and Table 1). The CD analysis of the CBM-CT fusion indicated the presence of α-helical secondary structure in addition to the predicted β-sheet conformation seen for the CBM construct alone.

**TABLE 1:** **Analysis of CD spectra using CDSSTR** Deconvoluted data used CDSSTR (48) and are represented as a percentage of total protein.

Sample	Helix	Strand	Turns	Unordered	NRMSD*^a^*
CBM-CT	40	20	16	24	0.015
CBM	5	44	23	28	0.019

*^a^* Normalized root mean square deviation (NRMSD) between experimental spectrum and the spectrum calculated using CDSSTR parameters.

##### Analysis of CBM Monosaccharide Binding

**FIGURE 6.**
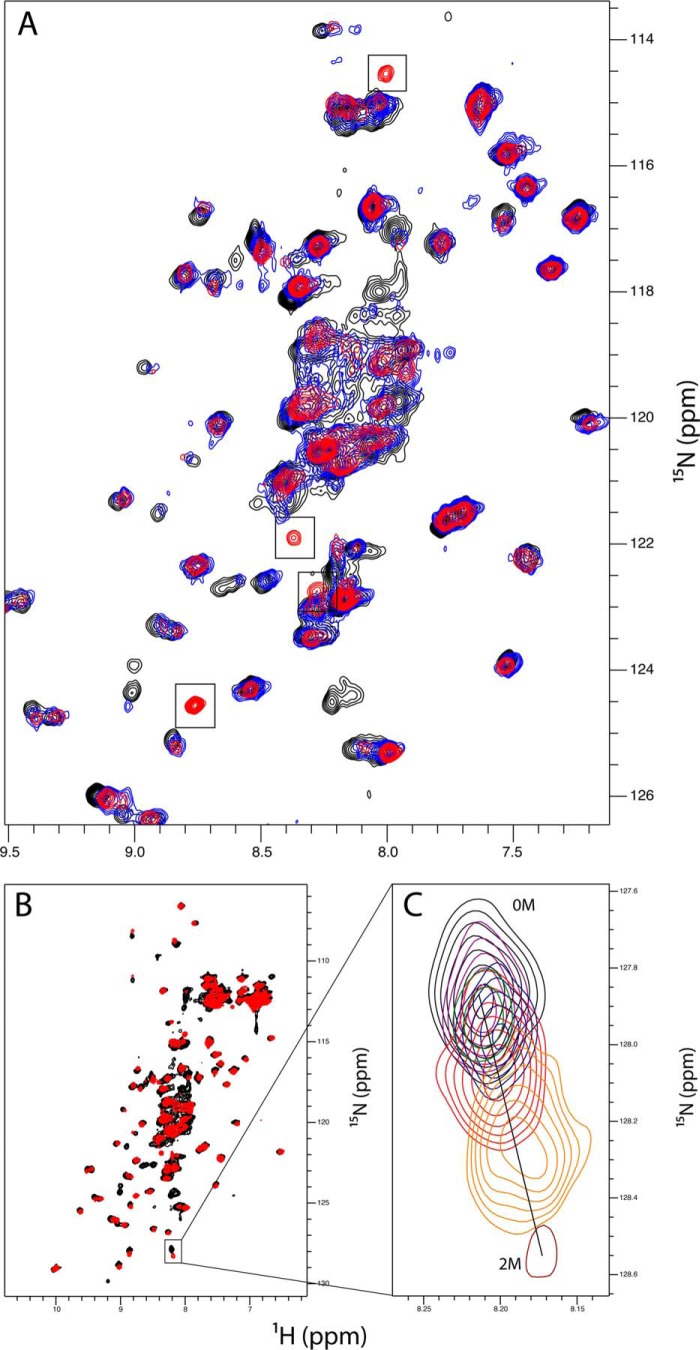
**NMR analysis of the CBM in the presence of monosaccharides.** A magnified region of the ^15^N HSQC spectra is shown for the untreated CBM (*black*) and the CBM titrated with 1 m d-galactose (*red*) or 1 m l-fructose (*blue*). The four peaks, which are unique to the d-galactose-treated sample, are indicated by *black boxes* (*A*). The complete ^15^N HSQC spectra for untreated CBM (*black*) and CBM with 1 m d-galactose (*red*) show nonspecific chemical shift perturbations upon the addition of d-galactose (*B*) with a magnification of one such example over the titration points of 0 mm, 62.5 mm, 125 mm, 250 mm, 500 mm, 1 m, and 2 m d-galactose.

To investigate potential carbohydrate binding capacity of the CBM, we used NMR spectroscopy to assess chemical shift perturbations of the protein spectra upon titration of monosaccharides (Fig. 6). Isotopically ^15^N-labeled protein was expressed in minimal media using an *E. coli* expression system. The labeled CBM protein could be purified in an identical manner to the unlabeled sample for subsequent use in NMR spectroscopic measurements. The CBM gave a dispersed ^1^H,^15^N HSQC spectrum indicative of a folded domain, in support of both the results of the CD experiments and the bioinformatics analysis.

A series of HSQC spectra was collected for the CBM in the presence of increasing concentrations of monosaccharides. When aligned against an untreated sample, chemical shift perturbations were apparent upon the addition of d-galactose. Considering the large molar excess of monosaccharide used in these experiments, l-fructose was used as a control to ascertain if these changes were specific to d-galactose. It was apparent that some of these perturbations to the spectra also occurred with titration of l-fructose. However, four distinct peaks appeared upon d-galactose titration, which were visible from the lowest titration point. These peaks did not appear in the l-fructose-treated sample and were not present in the untreated sample.

**FIGURE 7.**
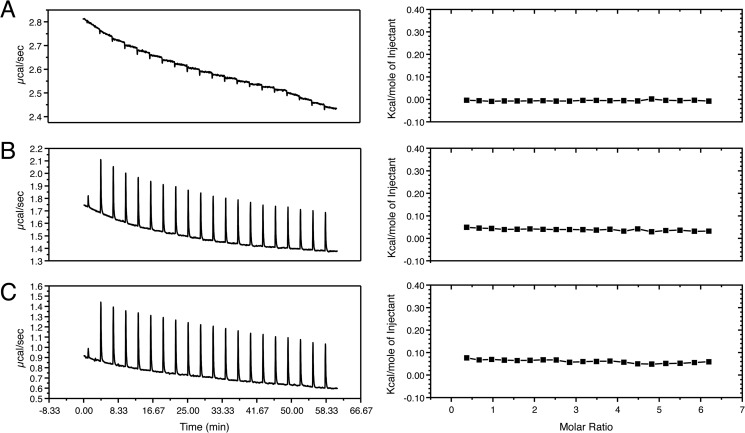
**Isothermal calorimetry titrations of the CBM with d-galactose.** The *left-hand panels* indicate the raw data collected during titration of the CBM with d-galactose. The *right-hand panels* show the corresponding enthalpy changes of the integrated peaks plotted as the change in kcal/mole of injectant (PBS or d-galactose) *versus* the molar ratio of CBM to d-galactose. The titration of d-galactose into PBS shows a negligible exothermic change in enthalpy attributable to the heat of dilution of the d-galactose solution into PBS (*A*). The titration of d-galactose into a solution of CBM gives endothermic changes in enthalpy at each injection point (*B*). The titration of PBS into a solution of CBM also shows endothermic changes in enthalpy at each injection point, comparable to those that occur upon the addition of d-galactose (*C*). None of the titrations give an isotherm curve indicative of a binding event (*right-hand panels*).

Isothermal calorimetry was used to further investigate the potential galactose binding ability of the CBM. Although an endothermic change in enthalpy was evident upon titration of galactose to the CBM, this change could be attributed to the heat of dilution of the CBM sample in the cell, which displayed a similar endothermic profile when PBS alone was added (Fig. 7).

##### C-terminal Deletion Impairs EndoS Activity

**FIGURE 8.**
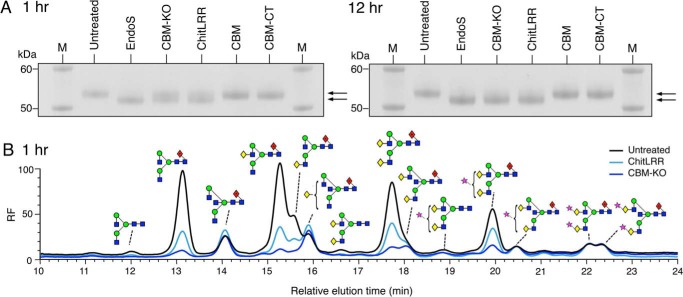
**Deletion of the CBM or C-terminal region of EndoS affects glycoside hydrolase activity against serum IgG.** SDS-PAGE analysis of the reaction components of enzymatic digest of serum IgG with EndoS or EndoS domains as denoted *above each lane* of the SDS-PAGE gel, where *M* indicates the molecular weight marker. The *left panel* shows the SDS-PAGE analysis after a 1-h incubation at 37 °C of IgG with the EndoS domains. The *right panel* shows the reactions analyzed in an identical manner after 12 h of incubation at 37 °C. The *arrows to the right of both panels* indicate the position of the upper (glycosylated) and lower (deglycosylated) bands representative of the heavy chain of serum IgG (*A*). HPLC analysis of residual serum IgG glycan species present after a 1-h incubation with either CBM-KO and ChitLRR. Chromatograms were normalized to the glycan peak at 22 min (*B*). *RF*, relative fluorescence units.

Considering the evidence for the presence of a CBM within EndoS, we sought to assess whether deletion of this region had an effect on EndoS activity. The EndoS truncations, CBM-KO and ChitLRR, were incubated with human serum IgG to assess their ability to cleave the glycans from the heavy chain of IgG. After incubation of the reaction at 37 °C, the components were analyzed by reducing SDS-PAGE. A molecular weight shift of the band representative of the IgG heavy chain was used to judge the glycosylation state of the IgG. After a 1-h incubation, a reduction in molecular mass (∼3 kDa) of the IgG heavy chain band could be seen for the EndoS-treated sample when compared with the untreated IgG sample. At this 1-h time point, both the CBM-KO and ChitLRR-treated samples revealed the presence of two bands for the IgG heavy chain (Fig. 8*A*, *left panel*). Glycan analysis of the upper band from these reactions (Fig. 8*B*) revealed that both CBM-KO and ChitLRR left a residual population of bisected glycans at this time point in addition to the di-sialylated glycans, which also remain uncleaved by full-length EndoS after 24 h (Fig. 3*A*). When compared with CBM-KO, ChitLRR left an additional population of complex glycans uncleaved after 1 h.

Upon longer incubation, it was apparent that these truncated forms of EndoS were able to produce an SDS-PAGE profile similar to full-length EndoS. This could be seen 12 h after the digestion was initiated (Fig. 8*A*, *right panel*). Here, a single band for the IgG heavy chain was present for both CBM-KO- and ChitLRR-treated samples, which ran at a lower apparent molecular mass compared with untreated IgG sample.

## DISCUSSION

### 

#### 

##### Glycan Analysis of IgG Fragments

EndoS is a glycoside hydrolase secreted by *S. pyogenes* during infection. In addition to its apparent contribution to immune evasion by *S. pyogenes*, it is of interest as a tool for treatment of immunological diseases (for review, see Ref. 13) as a transglycosidase (7) and as a tool for enhancing specific antibody-receptor interactions (5, 49). We aimed to investigate the activity of EndoS against IgG to better understand the interaction between these two molecules to maximize the potential of EndoS in these applications.

Initially, we sought to determine a minimal IgG substrate unit from which EndoS was able to cleave glycans and, as such, developed a number of mammalian expressed fragments of human IgG1. Fragments Fc, CH2-H and CH2 could all be expressed in the soluble fraction. All fragments were glycosylated, although the levels of glycan processing were different between all fragments. This suggested an effect of quaternary structure on glycan processing for these IgG fragments. In particular, the high proportion of digalactosylated glycans in the isolated CH2 fragment echoes that seen in previous analysis of truncated IgG domains of IgG expressed in Chinese hamster ovary cells (40). The presence of hybrid glycan species in the CH2-H fragment and in the CH2 fragment was indicative that these protein structures were changing the accessibility of the *N*-linked glycans to Golgi α-mannosidase II.

Using these glycosylated IgG fragments, we were able show that EndoS could cleave the glycans from each of these fragments including the isolated CH2 domain from IgG. In all cases, EndoS could cleave the majority of the glycans from each of the constructs, which suggested that the Fab region of IgG and the CH3 domains of IgG Fc were dispensable for EndoS activity against IgG. Looking at the CH2 domain in isolation, EndoS was again able to cleave glycans from this construct, which suggested that dimerization of the IgG Fc region as well as the hinge of IgG were not required for EndoS to deglycosylate IgG. Despite this, it appeared that the CH3 region and dimerization may influence the activity of EndoS against IgG. Looking at CH2-H and CH2 deglycosylation, it was apparent that more residual glycans were left on the protein scaffold after a 24-h incubation with EndoS when compared with Fc or serum IgG. However, this reduction in deglycosylation efficiency could be due to minor conformational changes or folding of the substrate due to expression of these domains in isolation. In addition, because the glycans were not of exactly the same composition on all expressed fragments, it was not possible to directly compare rates of deglycosylation. Furthermore, although complex bisected biantennary glycans could be cleaved by EndoS, it appeared that EndoS had reduced activity against these glycans compared with complex biantennary types. This was more apparent in the case of CH2-H and CH2 compared with Fc and IgG and was supportive of the notion that the CH3 and heavy chain dimerization may influence activity, respectively. EndoS was also less active against the bisected triantennary or tetraantennary glycan types present on CH2-H and CH2. In a recent study of human plasma-derived IgG, these glycans were not present (41), suggesting EndoS has developed some specificity for solely glycans expressed on human serum IgG.

In the context of *S. pyogenes* infection, IgG is likely to encounter a number of proteolytic and glycolytic enzymes including SpeB, IdeS, and EndoS2 (8, 9). The fact that EndoS appears to have activity on fragments of IgG, which do not contain either the hinge or Fab region, suggests that EndoS could continue to act on fragments produced by IdeS and SpeB cleavage. EndoS ability to act on these fragments may further compound the immune evasion strategy of *S. pyogenes*.

Putting this information together, it is of interest how EndoS maintains an apparent specificity for IgG given its ability to cleave the glycan from such a reduced protein scaffold. It is possible that its multidomain architecture may sterically hinder EndoS from cleaving other Ig substrates or simply that the subtle differences in the CH2 region of IgG alone are responsible for distinguishing this substrate of EndoS from other Ig classes.

##### EndoS Domain Architecture

To investigate components of EndoS that might be important for the IgG-EndoS interaction we analyzed the sequence of EndoS using the program FUGUE. FUGUE utilizes primary amino acid sequence to identify structurally homologous proteins and can be used to produce model outputs of tertiary structure based on alignments to recognize structural homologues (29). FUGUE was successful in recognizing the glycoside hydrolase family 18 chitinase domain in the N-terminal region of the protein. In addition, it was able to locate the LRR directly downstream of the chitinase domain. Both of these domains have been previously discussed in context of their homologous domains and will not be discussed here (10).

In addition to these two regions, a further region was identified to have homology to CBM family 32. The CBM32 family has been shown to bind monosaccharides and disaccharides found at the termini of mammalian *N*-linked glycans (for review, see Ref.^42^). The putative CBM from EndoS showed homology to the galactose binding domain of a sialidase from *Micromonospora viridifaciens* (43) and the galactose binding domain of galactose oxidase from *Dactylium dendroides* (44). Both of these CBM domains have been shown to bind galactose, as their name suggests, with mm affinity. This low affinity binding of CBMs to carbohydrate moieties is typical for this family of proteins (39). The function of this domain in these homologues is likely to aid avidity and selectivity of the enzyme. In the case of EndoS, a CBM could indeed aid the function of the enzyme and may be responsible for conferring specificity to IgG glycans. In addition to these two galactose binding domains, another homologue identified was the CBM32 domain of the endo-β-1,4-*N*-acetylglucosaminidase. Endo-β-1,4-*N*-acetylglucosaminidase is produced by the related bacteria *Streptococcus pneumonia*, which can cleave some oligomannose-type glycans, such as Man_5–4_GlcNAc_2_, and paucimannose-type structures such as Man_3_GlcNAc_2_ (45). A ligand for this CBM could not be identified, but structural features of the putative binding site suggested its function could involve both substrate capture and adhesion of *S. pneumonia* to host tissue via carbohydrates. Considering the role of these homologous proteins, it is suggested here that if EndoS does indeed contain a CBM, it is likely to aid in substrate recognition and enzyme avidity thus contributing to EndoS function.

Having identified this putative domain, we were able to clone and express a soluble form of the proposed CBM in isolation. Analysis by CD revealed the domain as containing primarily β-sheet structure with a large proportion of loops and turns, supportive of the bioinformatics assignment of this region as a CBM. In addition, analysis of the CBM by NMR gave a dispersed ^1^H,^15^N HSQC spectrum indicative of a folded domain. NMR titrations with monosaccharides indicated possible monosaccharide binding capabilities, manifested as the appearance of new peaks; however, isothermal calorimetry titration experiments could not detect measurable binding of d-galactose to the CBM assuming a *K_d_* of 1 mm. Although the d-galactose-induced peak appearances in the HSQC spectra are indicative of a specific interaction, the inability to detect an interaction via isothermal calorimetry titration suggests that any interaction is low affinity, as is typical of CBMs (39). Alternatively, these peaks may arise as an artifact of the large molar excess of d-galactose used in NMR titrations. However, considering these peaks were not evident in either the d-galactose only (data not shown) or the l-fructose CBM control experiments, it is conceivable the CBM can specifically bind monosaccharides, but with low affinity. Overall, further investigation is required to consolidate the observations seen in these experiments and to determine the ligands of the EndoS CBM.

##### C-terminal Deletion Impairs EndoS Activity

To assess the possible effects of the CBM on EndoS activity, we designed truncations where this region was deleted entirely (CBM-KO) and where both the CBM and CT regions were truncated (ChitLRR). These constructs could both be expressed and purified as soluble proteins. Upon deletion of either the CBM or the CBM and CT regions, EndoS exhibited a difference in activity against serum IgG when compared with full-length EndoS. Using SDS-PAGE to assess serum IgG digested with the various constructs, we found that where EndoS could produce a single band representative of the deglycosylated heavy chain of IgG, CBM-KO and ChitLRR produced two bands, indicative of both deglycosylated and glycosylated forms of IgG heavy chain present in the reaction mixtures. After further incubation, it was apparent that both CBM-KO and ChitLRR could deglycosylate IgG to a comparable level to that achieved by EndoS. HPLC analysis of the glycans, which remained on IgG after a 1-h incubation with these constructs, revealed residual populations comprising primarily bisected glycans. This observation perhaps indicates a role of the C-terminal region in IgG avidity, which allows these potentially more resilient glycoforms to be cleaved.

In conjunction with the analysis of the isolated CBM and CBM-CT domains, the results of these experiments suggest that the C-terminal region of EndoS contributes to its activity. This contribution is likely to occur via a CBM; however, it cannot be ruled out that the CT also contributes to EndoS function. Indeed, the data presented here suggest that the CT contributes further activity to EndoS based on the differences seen between the specificity and activity of CBM-KO and ChitLRR against serum IgG glycans.

It is possible that deletion of the CBM domain in CBM-KO could disrupt function due to a change in overall protein architecture rather than a loss of CBM function. However, considering the expressed CBM-KO and ChitLRR are both soluble and still exhibit glycoside hydrolase activity, it is suggestive of both constructs being correctly folded and that the CBM and CT region of EndoS do in fact have a role in activity against IgG.

Understanding the specificity and mechanism of any compound to be used for clinical applications is of upmost importance to safety and efficiency. Therefore, understanding the interaction of EndoS with IgG and the contributions of each required to elicit activity and specificity is of use if EndoS is to be used for the discussed applications. Here, we demonstrated that EndoS did not require the Fab or CH3 domains of IgG to exhibit glycoside hydrolase activity against the CH2 domain of IgG. We were also able to identify a CBM in the C-terminal region of EndoS that showed a potential for low affinity binding to d-galactose. Finally, we were able to show that deletion of the C-terminal region of EndoS, including this CBM, causes a reduction in activity of EndoS against serum IgG. Overall, these studies have contributed to the characterization of the important and useful interaction of EndoS with IgG.

## Footnotes

4 The abbreviations used are:

EndoS: endoglycosidase S
Chit: chitinase
CBM: carbohydrate binding module
CT: C-terminal region
Hex: hexose
HSQC: heteronuclear single-quantum coherence
LRR: leucine-rich repeat
PNGase F: peptide *N*-glycosidase F
Bis-Tris: 2-[bis(2-hydroxyethyl)amino]-2-(hydroxymethyl)propane-1,3-diol.
